# Supplementary material for: Volumetric non-invasive cardiac mapping for accessible global arrhythmia characterization
Source: Commun Med (Lond). 2026 Jan 13;6:263. doi: 10.1038/s43856-025-01332-5 (PMC13136336; doi:10.1038/s43856-025-01332-5)
Supplement: Supplementary file 1 — Supplementary information [file 43856_2025_1332_MOESM1_ESM.pdf]

# Supplementary Information for:

Volumetric Non-Invasive Cardiac Mapping for Accessible Global Arrhythmia Characterization

## Description of Supplementary Files

This document describes the contents of the Supplementary Data files accompanying the manuscript. The raw data supporting the findings of this study are provided in three Microsoft Excel files, renamed as Supplementary Data 1, 2, and 3.

### Supplementary Data 1

*Source data underlying Figure 4. Contains the sheets:*

- **CoarseMesh_Vertices**: Coordinates of the vertices for the inverse problem mesh (coarser than the simulation mesh).
- **CoarseMesh_Tetrahedra**: Vertex indices for each tetrahedron in the mesh.
- **Freewall_Simulation**: Activation times (ms) of the freewall ventricular ectopic simulation, projected onto the inverse problem mesh.
- **Freewall_Epicardial**: Activation times (ms) obtained from the epicardial reconstruction of the freewall ectopic beat.
- **Freewall_Source**: Activation times (ms) obtained from the volumetric reconstruction of the freewall ectopic beat.
- **Base_Simulation**: Activation times (ms) of the base ventricular ectopic simulation, projected onto the inverse problem mesh.
- **Base_Epicardial**: Activation times (ms) obtained from the epicardial reconstruction of the base ectopic beat.
- **Base_Source**: Activation times (ms) obtained from the volumetric reconstruction of the base ectopic beat.
- **Septum_Simulation**: Activation times (ms) of the septum ventricular ectopic simulation, projected onto the inverse problem mesh.
- **Septum_Epicardial**: Activation times (ms) obtained from the epicardial reconstruction of the septum ectopic beat.
- **Septum_Source**: Activation times (ms) obtained from the volumetric reconstruction of the septum ectopic beat.

### Supplementary Data 2

*Source data underlying Figure 5. Contains the sheet:*

- **FigX_BoxplotDistances**: Euclidean distances between the simulated and reconstructed origins across different groups for each reconstruction method.

**Supplementary Data 3**
*Source data underlying Figures 6 to 9 (Clinical Cases). Contains the sheets:*

- **LBBB_vertices**: Mesh coordinates used for the Left Bundle Branch Block (LBBB) patient.
- **LBBB_Tetrahedra**: Vertex indices for each tetrahedron in the LBBB patient mesh.
- **LBBB_ActivationTimes**: Reconstructed activation times (ms) for the LBBB patient.
- **PVC_vertices**: Mesh coordinates used for the Premature Ventricular Contraction (PVC) patient.
- **PVC_Tetrahedra**: Vertex indices for each tetrahedron in the PVC patient mesh.
- **PVC_ActivationTimes**: Reconstructed activation times (ms) for the PVC patient.
- **TV_vertices**: Mesh coordinates used for the Ventricular Tachycardia (VT) patient.
- **TV_Tetrahedra**: Vertex indices for each tetrahedron in the VT patient mesh.
- **TV_ActivationTimes**: Reconstructed activation times (ms) for the VT patient.
- **WPW_atria_vertices**: Atrial mesh coordinates used for the Wolff-Parkinson-White (WPW) patient.
- **WPW_atria_Tetrahedra**: Vertex indices for each tetrahedron in the atrial mesh of the WPW patient.
- **WPW_atria_ActivationTimes**: Atrial reconstructed activation times (ms) for the WPW patient.
- **WPW_ventr_vertices**: Ventricular mesh coordinates used for the WPW patient.
- **WPW_ventr_Tetrahedra**: Vertex indices for each tetrahedron in the ventricular mesh of the WPW patient.
- **WPW_ventr_ActivationTimes**: Ventricular reconstructed activation times (ms) for the WPW patient.
